# Autophagy and autophagic cell death in sepsis: friend or foe?

**DOI:** 10.1186/s40560-024-00754-y

**Published:** 2024-10-25

**Authors:** Toshiaki Iba, Julie Helms, Cheryl L. Maier, Ricard Ferrer, Jerrold H. Levy

**Affiliations:** 1 Department of Emergency and Disaster Medicine, Juntendo University Graduate School of Medicine, 2-1-1 Hongo Bunkyo-Ku, Tokyo, 113-8421 Japan; 2 Strasbourg University (UNISTRA); Strasbourg University Hospital, Medical Intensive Care Unit, NHC; INSERM (French National Institute of Health and Medical Research), UMR 1260, Regenerative Nanomedicine (RNM), FMTS, Strasbourg, France; 3 Department of Pathology and Laboratory Medicine, Emory University School of Medicine, Atlanta, GA USA; 4 Intensive Care Department, Hospital Universitari Vall d’Hebron Universitat Autònoma de Barcelona, Barcelona, Spain; 5 Department of Anesthesiology, Critical Care, and Surgery, Duke University School of Medicine, Durham, NC USA

**Keywords:** Autophagy, Sepsis, Mitochondria, Apoptosis, Necrosis, Mitophagy

## Abstract

In sepsis, inflammation, and nutrient deficiencies endanger cellular homeostasis and survival. Autophagy is primarily a mechanism of cellular survival under fasting conditions. However, autophagy-dependent cell death, known as autophagic cell death, is proinflammatory and can exacerbate sepsis. Autophagy also regulates various types of non-inflammatory and inflammatory cell deaths. Non-inflammatory apoptosis tends to suppress inflammation, however, inflammatory necroptosis, pyroptosis, ferroptosis, and autophagic cell death lead to the release of inflammatory cytokines and damage-associated molecular patterns (DAMPs) and amplify inflammation. The selection of cell death mechanisms is complex and often involves a mixture of various styles. Similarly, protective autophagy and lethal autophagy may be triggered simultaneously in cells. How cells balance the regulatory mechanisms of these processes is an area of interest that is still under investigation. Therapies aimed at modulating autophagy are considered promising. Enhancing autophagy helps clear and recycle damaged organelles and reduce the burden of inflammatory processes while inhibiting excessive autophagy, which could prevent autophagic cell death. In this review, we introduce recent advances in research and the complex regulatory system of autophagy in sepsis.

## Introduction

Sepsis is a severe and life-threatening condition caused by a systemic inflammatory response to infection. The cascades of inflammation and over-activated or suppressed immune reactions facilitate the development of deadly organ dysfunction and death. Cell death and its rescue system play major roles in determining the patients’ outcomes [1]. Autophagy is primarily an adaptive response to protect cells from environmental stresses, especially nutrient defects [2]. However, autophagy is not always cytoprotective and host-defensive; it can result in unfavorable outcomes if autophagy is excessive or inadequate. For instance, autophagic cell death is inflammatory and contributes to tissue damage [3]. This review introduces a detailed story of the relationship between autophagy, autophagic cell death, and cell death in sepsis.

## Types of autophagy

Autophagy is a cellular process involving lysosomal degradation and recycling of organelles, proteins, and other cellular components. It is essential for maintaining cellular homeostasis and responding to infection-induced stress. The term “autophagy” literally means “self-eating” and usually be classified into three types [4].

Macroautophagy is the most well-studied type, often referred to as autophagy. It involves forming a double-membrane structure called an autophagosome that engulfs cytoplasmic components non-selectively and then fuses with a lysosome to degrade the contents [5]. Recycled contents are used as nutrients and building blocks for organelles, aiding in cell survival. For example, autophagy plays a role in enhancing neutrophil phagocytosis during sepsis.

Microautophagy involves the direct engulfment of cytoplasmic materials by the lysosome through invagination of the lysosomal membrane [6].

Chaperone-mediated autophagy is a selective degradation of specific proteins recognized by chaperones and translocated directly into the lysosome [7].

Another classification divides autophagy into two categories based on cargo selectivity: selective autophagy and nonselective autophagy. Nonselective autophagy refers to the bulk transport of organelles or other components to lysosomes, while selective autophagy involves the degradation of specific substrates, such as mitochondria, in a process known as mitophagy [8] (Table 1).

**Table 1:** Types of the autophagy

Non-selective autophagy	Macroautophagy	Double-membrane autophagosome engulfs cytoplasmic components and degrades them by fusing with the lysosome
	Microautophagy	Direct engulfment of cytoplasmic materials by the lysosome through invagination of the lysosomal membrane
Selective autophagy	Chaperone-mediated autophagy	Specific cytoplasmic proteins recognized by chaperones are translocated into the lysosome and degraded
	Mitophagy	Damaged mitochondria are recycled by degradation, adding proteins, lipids, and other components

The cell death styles are classified into autophagic cell death (or autophagy-dependent cell death), which is induced by the autophagy mechanism, and autophagy-mediated cell death, which is mediated by autophagy but depends on different types of cell death mechanism [9].

Autophagy, dysregulated autophagy, autophagic cell death, and autophagy-mediated cell death play pivotal roles in the pathophysiology of various diseases, including infections, sterile inflammations, neurodegenerative diseases, and malignancies [10]. In sepsis, both autophagic cell death and autophagy-mediated cell death amplify host inflammatory responses and play a significant role in the pathophysiology.

## Mechanism of autophagy

**Fig. 1:**
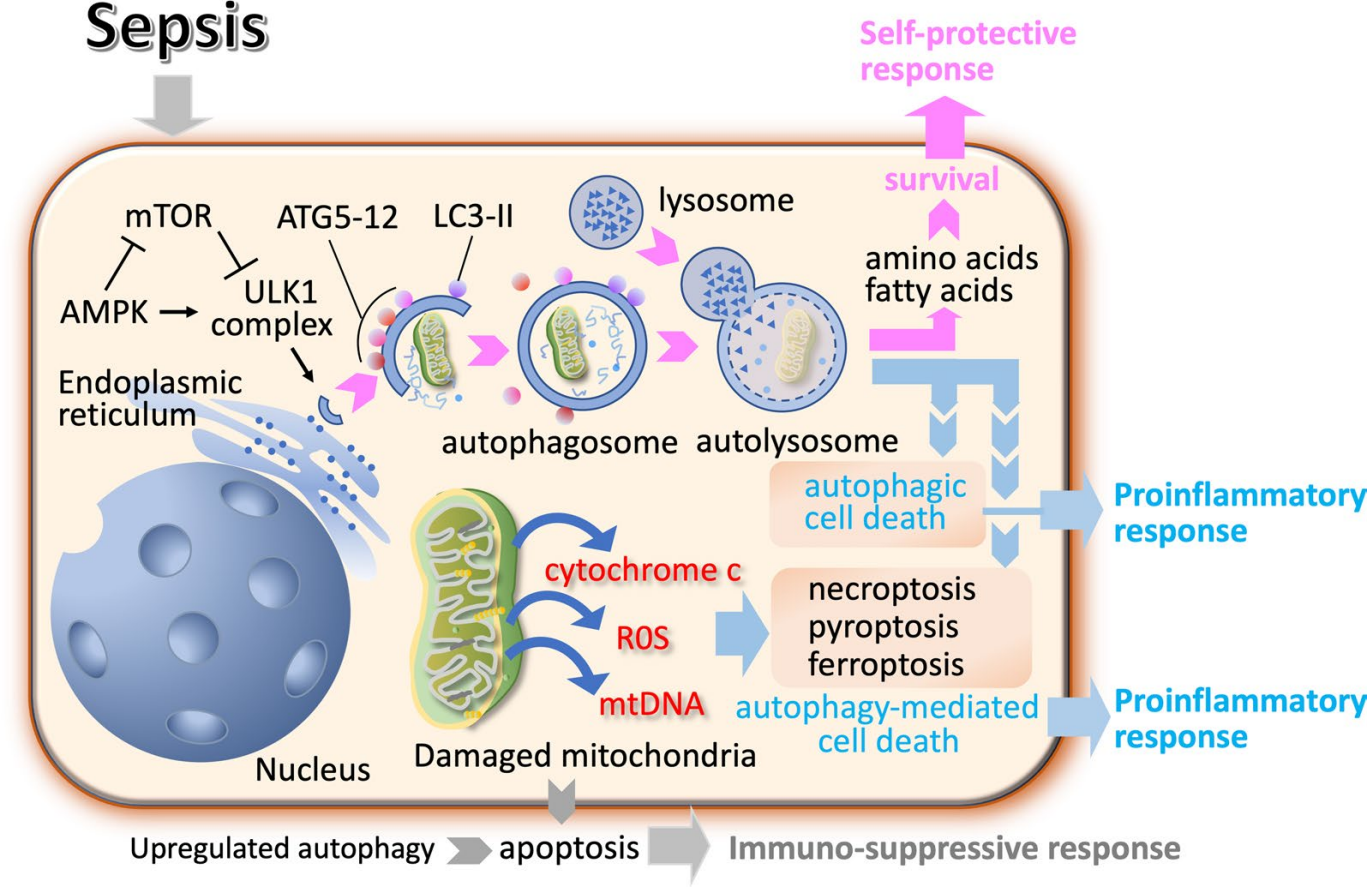
Mechanism of macroautophagy. Autophagy plays a crucial role in regulating inflammation during sepsis. In sepsis, adenosine monophosphate (AMP)-activated protein kinase (AMPK) inhibits mTOR (mammalian target of rapamycin), which weakens the inhibitory effect of mTOR complex 1 on the formation of the ULK1 (Unc-51-like kinase 1) complex, thereby promoting the production of autophagic vesicles. The polymer complex autophagy-related protein (Atg)–Atg12–Atg16L is formed by a series of Atg5, Atg12 and ATtg16L actions, and then, the polymer complex is fused with autophagic vesicles. Microtubule-associated protein 1 light chain 3 (LC3) is inserted into autophagosomes through a series of reactions. The cysteine protease Atg4 cleaves LC3 into LC3-I, which is then processed by Atg3, Atg7 and phosphatidylethanolamine to form LC3-II. Subsequently, LC3-II is inserted into the autophagosomes. Autophagosomes and lysosomes fuse to form autolysosomes. The regulation of inflammation by autophagy is complex and condition-dependent. While autophagy typically promotes cell survival, dysregulated autophagy can lead to pro-inflammatory cell death

Among the three autophagy types, the mechanism of macroautophagy (hereafter referred to as autophagy) is the best known. Under stress conditions, mTOR (mammalian target of rapamycin) is inhibited, leading to the activation of the ULK1 (Unc-51-like kinase 1) complex (Fig. 1). Following ULK1 complex activation, the formation of the phagophore is initiated [11]. Autophagy-related proteins (Atgs), including Atg5, Atg12, and Atg16L1, form a complex that aids in elongating the phagophore membrane [12]. Following that, microtubule-associated protein 1 light chain 3 (LC3) is inserted into autophagosomes through a series of reactions. Then, the cysteine protease Atg4 cleaves LC3 into LC3-I, which Atg3, Atg7, and phosphatidylethanolamine the process to form LC3-II. Subsequently, LC3-II is inserted into the autophagosomes [13]. The phagophore membrane then closes around the cargo, forming a double-membrane autophagosome. Finally, the mature autophagosome fuses with a lysosome, forming an autolysosome [14]. The inner membrane and contents of the autolysosome are degraded by lysosomal hydrolases, and the resulting macromolecules, such as amino acids, fatty acids, and sugars, are released back into the cytoplasm for reuse by the cell [15]. Autophagy is regulated by nutrient levels, energy status, and growth factors that modulate mTOR activity. The AMP-activated protein kinase (AMPK) pathway also senses cellular energy levels and can activate autophagy by inhibiting mTOR and directly phosphorylating ULK1 [16]. Other signals, such as hypoxia, reactive oxygen species (ROS), and intracellular calcium levels, can also influence autophagy activity. Autophagy is significantly involved in enhancing neutrophil functions during sepsis, aiding in pathogen clearance, and modulating immune responses.

## Roles of autophagy

**Fig. 2:**
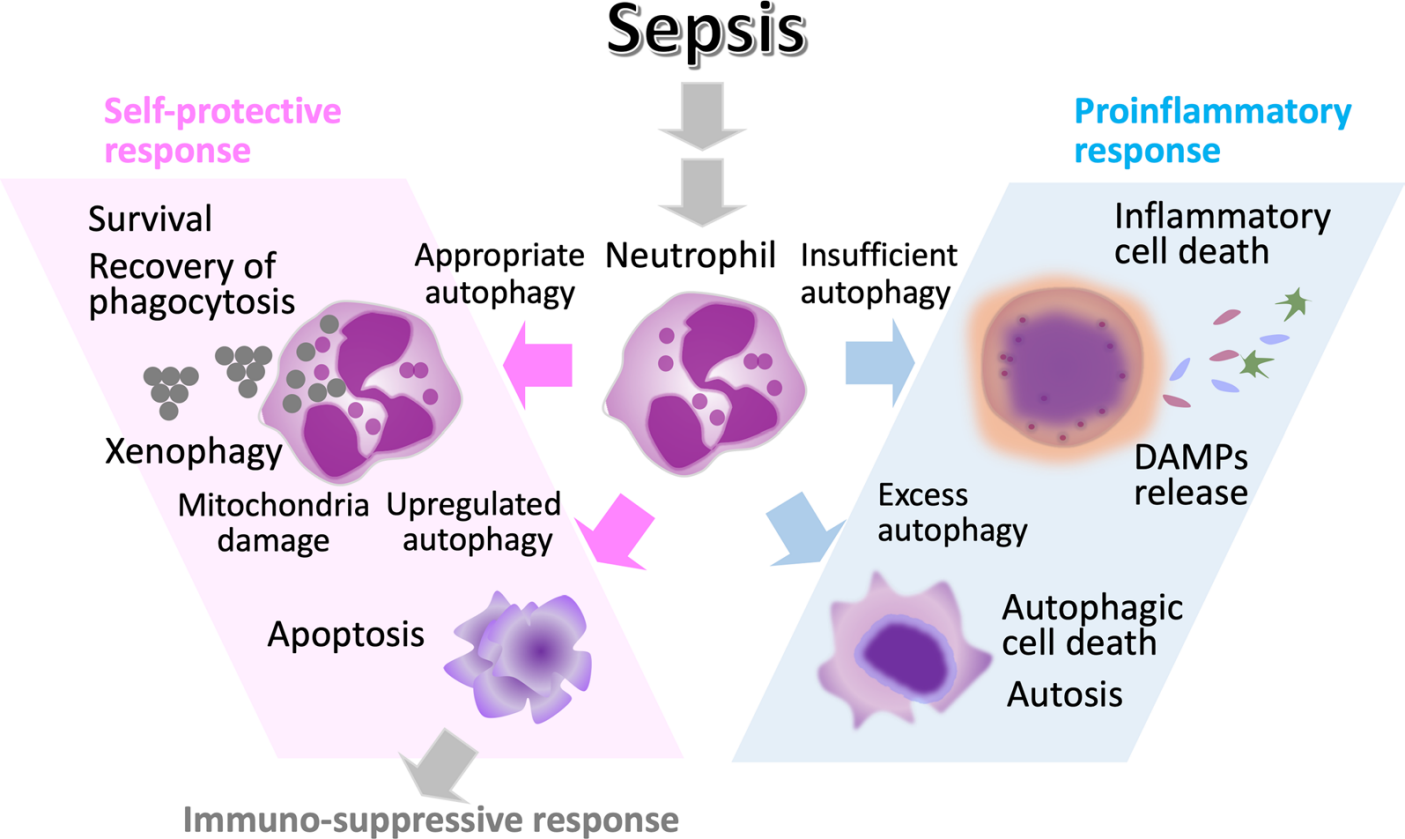
Regulation of autophagy and cell deaths. Autophagy is a mechanism that supports cellular survival. However, if the stress is too severe, insufficient or inadequate autophagy can lead to autophagy-mediated cell death. In cases of extremely severe stress like sepsis, excessive or dysregulated autophagy can induce inflammatory cell deaths such as autophagic cell death or autosis. However, the exact relationship between autophagy and various forms of cell death has yet to be clarified

Autophagy is a cellular process that involves the degradation and recycling of unnecessary or damaged organelles, misfolded proteins, and cellular debris [17]. Cells activate it as a survival mechanism in response to stress, nutrient deprivation, and organelle damage. On the other hand, excess autophagy is known to result in cell death [9]. Autophagy modulates neutrophil-mediated inflammation by controlling key functions, such as phagocytosis and ROS generation, which are crucial for fighting infections in sepsis [18] (Fig. 2).

### Promotion of cell survival

As repeatedly mentioned, the primary objective of autophagy is to promote cell recovery by maintaining cellular homeostasis and energy balance, particularly under stress and nutrient deprivation [19]. Mitochondria, the organelles acting as powerhouses and essential energy suppliers, are major autophagy targets. Sepsis induces hyperinflammation, hypoxia, and a catabolic state, leading to mitochondrial damage. This damage, in turn, causes cellular injury by improperly producing ROS and releasing cytochrome c and mitochondrial DNA. Both selective and bulk autophagy function to clear damaged mitochondria, preventing the accumulation of dysfunctional mitochondria that deteriorate cellular function and induce cell death [20]. In this context, autophagy not only recycles intracellular components to provide essential nutrients and energy but also contributes to scavenging toxic substances.

### Modulation of inflammation

Neutrophils are front liners of the host defense against microbial invasion. Autophagy is essential for maintaining neutrophil homeostasis and functions such as phagocytosis, degranulation, ROS production, and cytokine release, which are vital for immune defense during sepsis [18]. However, if these responses are excessive, they can be harmful to the host.

On the contrary, autophagy can suppress excessive inflammation by degrading proinflammatory cytokines, including interferon (IFN)-γ, tumor necrosis factor (TNF)-α, and interleukin (IL) family (e.g., IL-1α, IL-1β, IL-33, and IL-36) involved in the inflammatory response [21, 22]. In addition, the autophagic degradation of NLRP3 (NOD-, LRR- and pyrin domain-containing protein 3) inflammasome and its activators, such as intracellular DAMPs and cytokines can reduce inflammasome activation and mitigate cell death by pyroptosis [23, 24].

### Xenophagy

Autophagy functions not only to eliminate self-organelles and proteins but also to remove non-self-pathogens, thereby playing a critical role in host defense against infections. Upregulated intracellular degradation of pathogens reduces the pathogen load, and this pathogen-lytic process is known as xenophagy. Xenophagy is a type of selective macroautophagy specifically used for the sequestration and degradation of pathogens within autophagosomes [25]. Through xenophagy, cells can identify, isolate, and digest pathogens, preventing their replication and spread.

Stimulator of interferon genes (STING) is a key protein in the innate immune response, particularly in detecting non-self-cytosolic DNA. During infections, autophagy can target intracellular pathogens for degradation, while STING activation can enhance the immune response against those pathogens [26]. STING-dependent signaling network is implicated in sepsis by regulating autophagic degradation of pathogens and induction of various cell death modalities [27]. The coordination between these processes ensures an efficient defense mechanism. Autophagy regulates the STING pathway by degrading activated STING, thus controlling the duration and intensity of the immune response [28]. This helps prevent excessive inflammation and potential autoimmunity, which leads to balancing the clearance of cellular debris and pathogens with the activation of appropriate immune responses.

### Regulation of cell survival and death

**Fig. 3:**
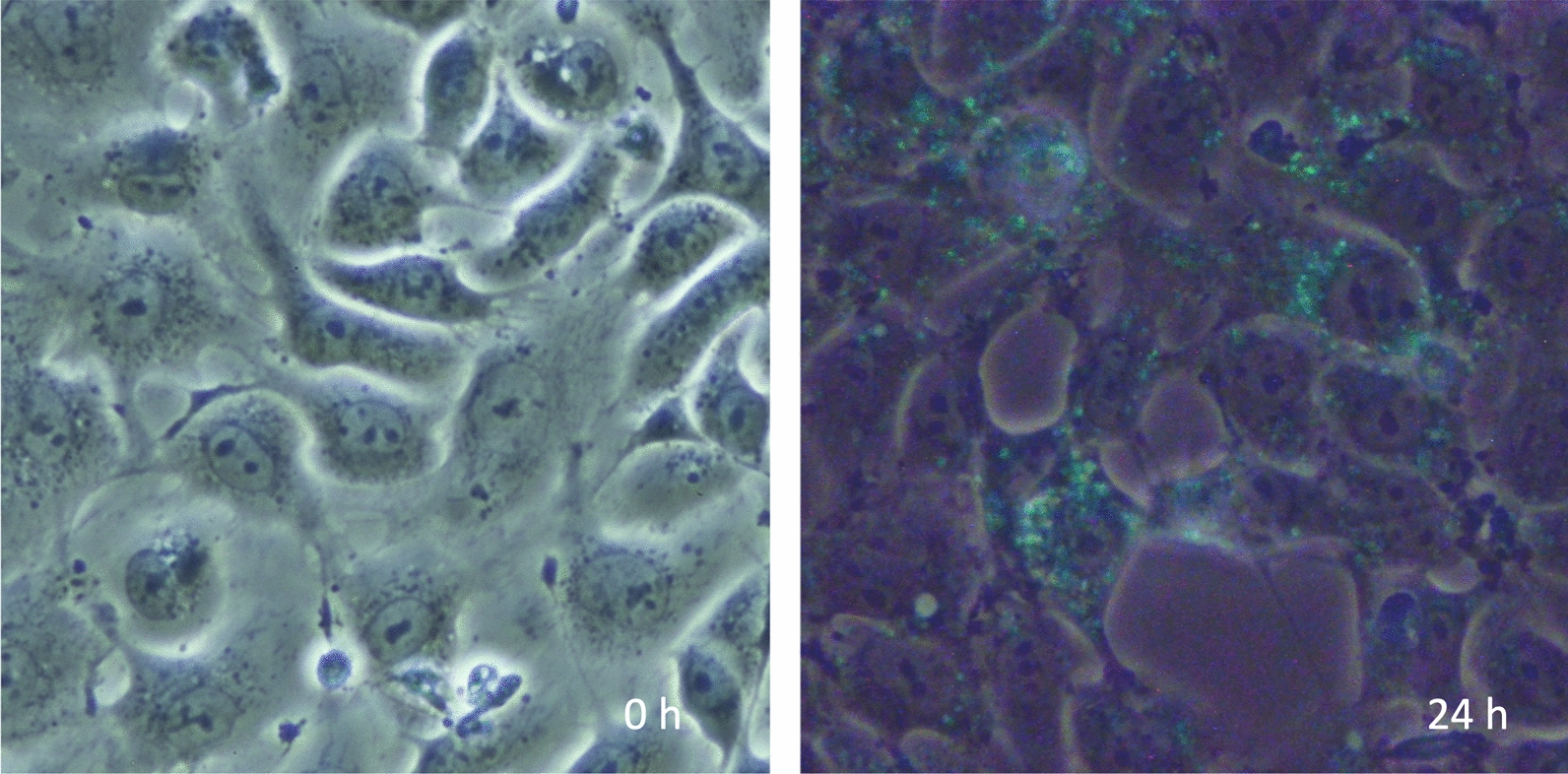
Autophagy of the damaged endothelial cells. The image shows vascular endothelial cells cultured without fetal bovine serum and subjected to histone H3, which induces endothelial damage and autophagy. The left panel, a phase-contrast view, demonstrates confluent cells. The right panel shows cell shrinkage and increased space between the cells. Autophagy is detected by monodansylcadaverine (MDC, green)

**Fig. 4:**
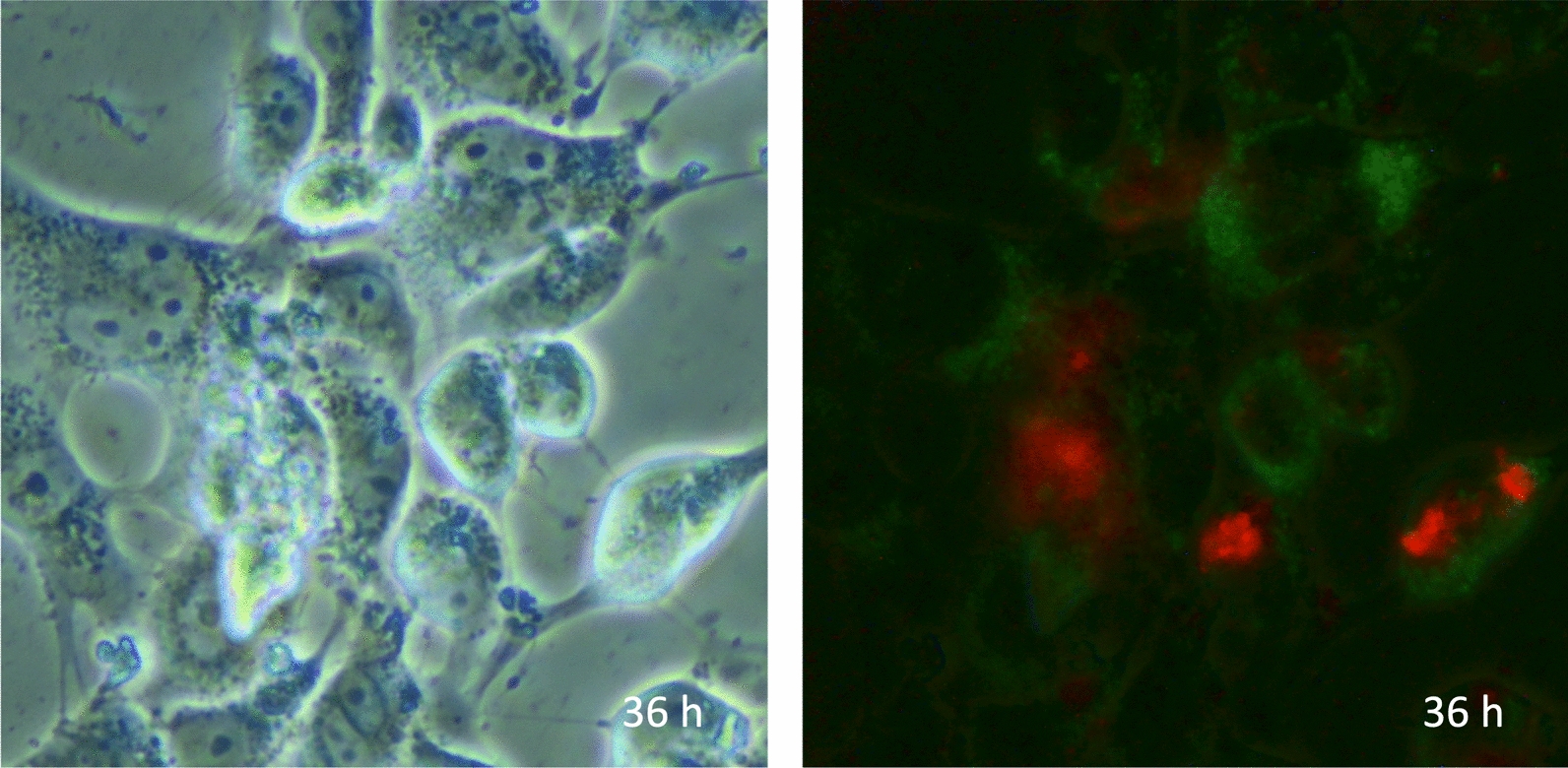
Autophagy of necrotic and survived endothelial cells. The image shows vascular endothelial cells cultured without fetal bovine serum and subjected to histone H3 for 36 h. The left panel, a phase-contrast microscopic view, demonstrates cells. In the right panel, autophagy is detected by monodansylcadaverine (MDC, green). The nuclei of necrotic cells are stained by propidium iodide (PI, red)

Although autophagy works for survival, cell death is inevitable if essential cellular components are degraded or sufficient nutrients are not provided. In this context, autophagy induces passive cell death, i.e., necrosis that results in inflammation (Figs. 3 and 4). Meanwhile, autophagy also leads to programmed cell death. In sepsis, apoptosis of macrophages increases due to upregulated autophagy, leading to immune suppression [22]. On the contrary, Park et al. [29] reported increased autophagy induction in neutrophils derived from septic patients and increased neutrophil cell death with neutrophil extracellular traps (NETs) ejection. In the latter case, autophagy-mediated cell death is considered an inflammatory cell death [30]. Both protective autophagy and lethal autophagy may be triggered simultaneously in sepsis, and how cells balance the regulatory mechanisms is still an open question [31]. The selection of cell death style may vary among cell types, inflammation severity, and infection phase. Liu et al. [32] performed an RNA sequence and electron microscopic examination to study alterations in immune cells in peripheral blood mononuclear cells in sepsis. The results showed signs of mitochondrial damage, autophagosomes, and cell surface pore-forming were more pronounced in severe sepsis. In addition, single-cell RNA sequencing revealed that cell death occurred mainly in myeloid cells rather than lymphocytes, and cell death patterns of destructive necrosis and pyroptosis were predominant in neutrophils, and apoptosis, ferroptosis, and autophagic cell death with less damage to the surroundings were predominant in monocytes. Autophagy-mediated cell deaths are introduced in the next section.

## Autophagy-mediated cell deaths

Various types of cell death, including accidental necrosis, programmed antiinflammatory apoptosis, programmed inflammatory necroptosis, pyroptosis, and ferroptosis, are induced in sepsis. Autophagy is responsible for avoiding and inducing cell deaths; however, the causal relationship between autophagy and various forms of cell death has yet to be clarified [33].

### Apoptosis

Apoptosis, or type I programmed cell death, is a controlled form involving activating specific signaling pathways and systematically dismantling cellular components. An increase in apoptosis is observed in various cells in sepsis, and apoptotic cell death is most prominently seen in immune cells such as monocytes, granulocytes, and lymphocytes. Too much apoptosis in leucocytes can impair the immune response and contribute to immunoparalysis, and the upregulation of autophagy to avoid apoptosis is favorable in sepsis [34]. Carchman et al. [35] reported heme oxygenase-1-mediated autophagy protects against hepatocyte cell death in a sepsis model. Meanwhile, insufficient or suppressed autophagy in sepsis tends to increase apoptosis. Lin et al. [36] reported down-regulation of autophagy in T-lymphocytes, resulting in enhanced apoptosis induction and decreased survival in sepsis. An increase in apoptotic cell death is permissive as long as the inflammation is under control; however, as the infection progresses, excess apoptosis should be avoided to combat infection [37].

### Necrosis

Necrosis, a type III cell death, is a non-programmed inflammatory cell death. DAMPs that amplify inflammation and tissue damage in sepsis increase following necrosis [38]. Under stress, even if apoptosis is initiated, cells eventually fall into necrosis due to the lack of energy required to complete the apoptotic process, and autophagy is expected to rescue cells from detrimental necrosis [39]. Autophagy is upregulated in sepsis to avoid necrotic cell death. Hsiao et al. [40] demonstrated the elevation of lipidated LC3-II, a marker of autophagy, in an animal model of sepsis-induced acute kidney injury. In addition, the knockdown of Atg7 exacerbated cell death, whereas rapamycin, an autophagy inducer, diminished cell death. The authors concluded that autophagy prevents cell death and mitigates proximal tubular cell injury in sepsis.

### Programmed inflammatory cell deaths

Other than necrosis, programmed inflammatory cell deaths such as necroptosis, pyroptosis, and ferroptosis play critical roles in driving inflammation, tissue damage, and organ dysfunction in sepsis [41].

Necroptosis is a caspase-independent form of programmed cell death mediated by receptor-interacting protein kinases (RIPK1 and RIPK3) and mixed lineage kinase domain-like protein (MLKL) [42]. Activation of these proteins leads to the formation of the necrosome, which translocates to the cell membrane and forms pores, causing membrane rupture and the release of cellular contents, including DAMPs [43]. Mitochondrial damage has been known to initiate necroptosis via the generation of ROS, leading to the release of DAMPs, mitochondria DNA, and other mitochondrial components [44]. Therefore, the degradation of damaged mitochondria by autophagy upregulation is necessary to avoid necroptosis.

Pyroptosis is another inflammatory cell death mediated by NLRP3 inflammasomes, multiprotein complexes that activate caspase-1, and a series of caspase pathways. Activated caspase-1 cleaves gasdermin D, forming pores in the cell membrane, causing cell lysis [45]. NLRP3 inflammasome, a multiprotein complex, is also involved in activating gasdermin D [46], releasing proinflammatory cytokines, such as IL-1β, IL-18, and DAMPs. Pyroptosis contributes to the massive inflammatory response in sepsis by releasing proinflammatory cytokines and DAMPs, which induce autophagy. In sepsis, the excessive production of cytokines can lead to heightened autophagic activity, potentially reaching a level where it induces harmful autophagic cell death [47]. Activated autophagy efficiently suppresses the above process and contributes to quenching excessive inflammation.

Ferroptosis is also a programmed inflammatory cell death characterized by lipid peroxide and free iron accumulation [48]. Autophagy can induce ferroptosis by degrading ferritin, which releases free iron and increases the cellular iron pool [49]. Conversely, autophagy protects cells by removing damaged mitochondria and reducing ROS production, thereby preventing ferroptosis [50]. In conclusion, autophagy plays a complex and crucial role in the regulation of necroptosis, pyroptosis, and ferroptosis during sepsis, influencing both the inflammatory response and cell survival.

## Autophagic cell death

Autophagic cell death, characterized by the extensive presence of autophagosomes and autolysosomes within the dying cell, is also known as type II programmed cell death. However, the classification of autophagic cell death as an independent cell death style is still a topic of debate among researchers. A distinct category for autophagic cell death is unlikely as autophagy is usually seen in each cell death style [51]. Unlike other programmed cell deaths, there is no specific pathway in autophagic cell death, and its overlap with other cell death pathways supports the idea that autophagic cell death is not an independent cell death style. It is claimed that the presence of autophagic markers in a cell undergoing death does not necessarily equate to autophagic cell death by itself [52]. Perhaps the boundaries between autophagic cell death and other types of cell death are blurred, and they may share common signaling pathways and regulatory mechanisms.

Regardless of whether autophagic cell death is an independent category, excessive autophagy is involved in various types of cell death in sepsis, exacerbating inflammation. In addition, if cells die through autophagy, they can release DAMPs into the extracellular space. DAMPs include high mobility group box 1 (HMGB1), adenosine triphosphate (ATP), and mitochondrial DNA. These DAMPs can trigger and amplify the inflammatory response by activating pattern recognition receptors (PRRs) on immune cells [53]. Autophagic cell death can increase during sepsis, although its role is complex and context-dependent. It should be noted that while autophagy is generally anti-inflammatory and cell-protective, autophagic cell death is proinflammatory and detrimental.

Autosis is a gene-dependent form of cell death mediated by the Na^+^ K^+^-ATPase pump. High levels of cellular autophagy characterize it and lead to cellular damage and death. This process occurs in response to autophagy-inducing peptides and severe cellular stress, such as extreme starvation. Autosis features unique characteristics that distinguish it from general autophagic cell death, driven by the accumulation of autophagic vesicles [54]. The presence of autosis suggests that autophagy is not merely a rescue system but actively contributes to cell killing in specific situations. Excessive autophagy in septic conditions has been shown to contribute to cell death, particularly in vital organs like the heart and liver, indicating that dysregulated autophagy during sepsis can lead to autosis and worsen organ failure [55].

### Trigger of autophagic cell death

Under conditions of excessive stress or damage, the capacity of autophagy can be overwhelmed or dysregulated, leading to autophagic cell death. The balance between autophagic cell death and apoptosis is tightly regulated in this context. The signaling pathways, such as the mTOR and AMPK pathways, modulate autophagy in response to cellular energy status and stress [56]. Proteins like Bcl-2 (B-cell/CLL lymphoma 2) can inhibit apoptosis and autophagy, highlighting the interconnected regulation of these processes [57]. It is unclear whether cells intentionally induce death by regulating autophagy, but autophagy is a determinant of cell fate. Dysregulated autophagy is induced by severe stress like sepsis can lead to cell death. Another key regulator is mitochondria and damaged mitochondria can release factors that trigger apoptosis, other programmed inflammatory cell deaths, and autophagic cell death [58]. The maintenance of mitochondrial function involves interrelated processes that collectively balance cellular homeostasis and respond to cellular stress. The choice of autophagic cell death and apoptosis determines cell fate, with mitochondria playing a pivotal role in energy production and cell death pathways regulation [59].

### Crosstalk between autophagy and inflammation

In sepsis, a feedback loop exists between autophagic cell death and inflammation. Inflammatory signals can induce autophagic cell death, and the resulting release of DAMPs from dead cells can further drive inflammation, creating a self-amplifying cycle that exacerbates tissue damage and organ dysfunction.

On the other hand, autophagy can remove NLRP3 inflammasome activators, such as intracellular DAMPs, NLRP3 inflammasome components, and cytokines, to decrease inflammasome activation and the inflammatory response. Dysfunction of autophagy leads to hyperinflammation due to the excessive activation of the NLRP3 inflammasome, thereby acting as a major suppressor of inflammation. On the contrary, inflammasome pathways can regulate autophagy, which maintains homeostasis and prevents detrimental inflammation [24]. The mechanism of balancing physiological autophagy and pathogenic autophagic cell death is a key research focus.

## Maintenance of mitochondria

Mitochondria are essential organelles for cellular survival, and autophagy functions to restore mitochondria. Mitochondrial function is significantly disrupted during sepsis, with increased oxidative stress and nitric oxide production contributing to mitochondrial damage [60]. Non-selective macroautophagy and selective mitophagy are fundamental systems for recycling mitochondria. Besides autophagy, alternative systems such as microvesicles and tunneling nanotubes aid the survival of neighboring or distant cells. In both systems, the primary cargo is mitochondria, but other organelles and proteins can also be transported.

### Mitophagy

As demonstrated, mitochondria are essential organelles responsible for energy production but also generate cytotoxic substances [61]. When mitochondria are damaged or dysfunctional, they generate excessive ROS, leading to cellular damage and death. Kaushal et al. [62] reported that while autophagy can involve the nonselective sequestration of cargo, it can also facilitate the selective degradation of damaged organelles, particularly mitochondria. Mitophagy is a selective form of autophagy that specifically targets damaged or superfluous mitochondria for degradation. This mechanism ensures mitochondrial quality control by removing damaged mitochondria, preventing the accumulation of dysfunctional mitochondria, and maintaining cellular homeostasis. Mitophagy renews mitochondrial components by adding protein and lipids through biogenesis, resulting in mitochondrial turnover [63].

Multiple mechanisms can induce mitophagy, and phosphatase and tensin homologue (PTEN)-induced putative kinase 1 (PINK1)–Parkin pathway, which regulates ubiquitin-dependent mitophagy, is best known [64]. In healthy conditions, PINK1 is transported to the inner mitochondrial membrane and cleaved and degraded by the ubiquitin–proteasome system [65]. When mitochondria are damaged, and the inner membrane is lost, PINK1 is stabilized on the outer mitochondrial membrane and activated through auto-phosphorylation, promoting Parkin recruitment. Parkin then poly-ubiquitinates other outer membrane proteins, which are subsequently phosphorylated by PINK1 [66]. This ubiquitin-dependent pathway and other mechanisms mediate the damaged mitochondria elimination.

It is suggested that mitophagy plays a critical role in sepsis by maintaining mitochondrial quality and controlling the immune response. Besides, autophagy is protective in preventing sepsis-induced organ failure, including in the kidneys and lungs, by clearing damaged mitochondria and reducing oxidative stress [63].

### Mitovesicles

**Fig. 5:**
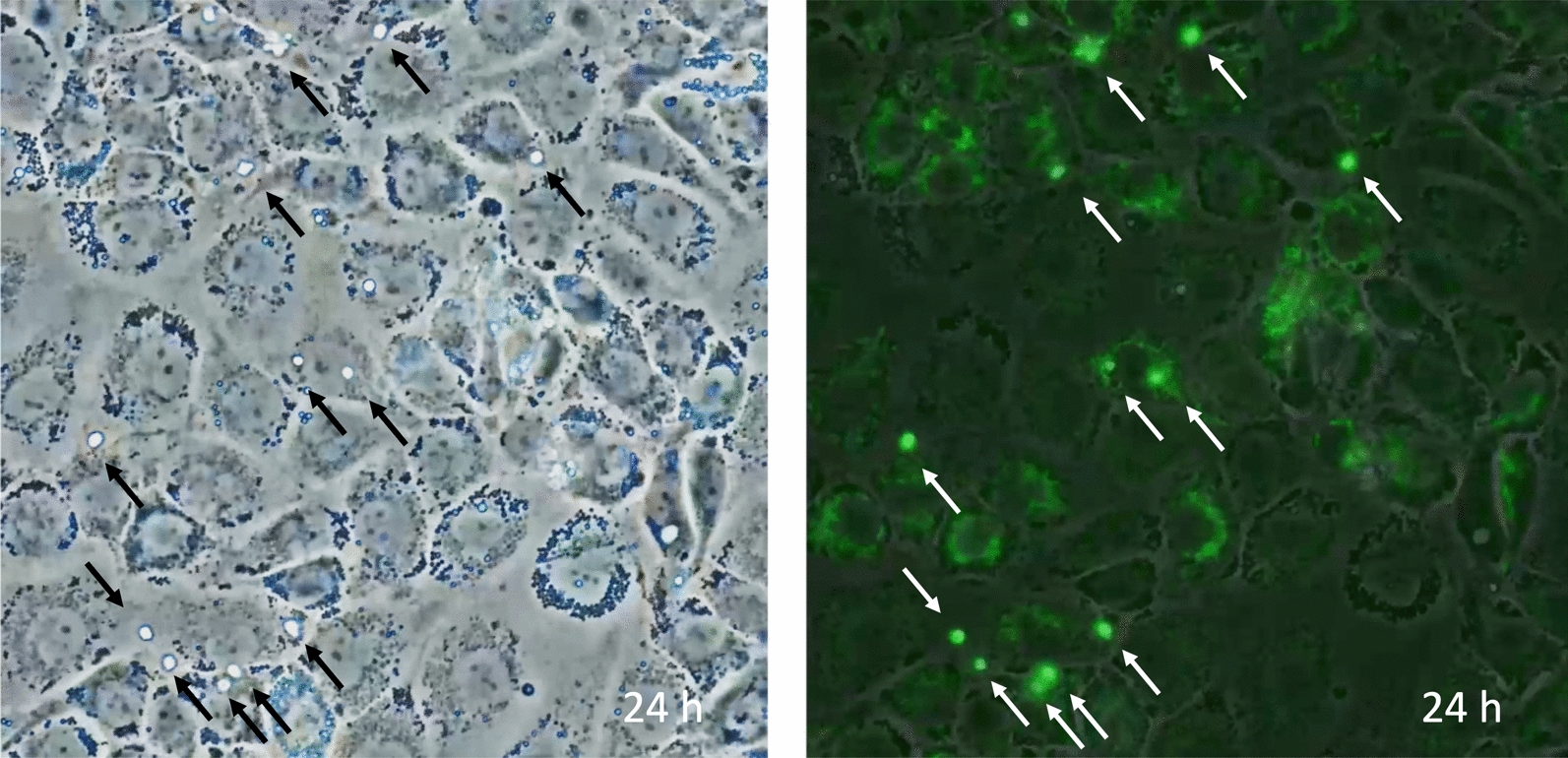
Mitochondria including microvesicles (Mitovesicles). The image shows cultured endothelial cells exposed to serum derived from *E. coli*-induced septic rats. The cells are observed and video-recorded under a microscope. After 24 h, microvesicles (black arrows) are released and float in the culture medium (left panel, confocal view). Mitochondria in the vesicles (mitovescles, white arrows) are stained with FITC (right panel, immunofluorescent view)

Extracellular vesicles, cellular fragments released by cells, play pivotal roles in physiological and pathological processes such as waste removal, cell-to-cell communication, and transport of protective or pathogenic material into the extracellular space [67]. The cargos that are carried by extracellular vesicles define their biological activities. Intercellular transfer of mitochondria and mitochondrial components through extracellular vesicles and extracellular vesicle-mediated transfer of mitochondria and its contents alter metabolic and inflammatory responses [68] (Fig. 5).

Thomas et al. [69] reported that mesenchymal stromal cells can rescue injured cells by donating mitochondria via microvesicles, restoring mitochondrial function in the recipient cells and preserving cell viability. Fluorescent nanoparticle tracking, immunoblotting, and flow cytometry revealed that mitochondrial cargoes are abundant across all extracellular vesicle size populations, with mitovesicles being among the largest extracellular vesicles. Polarization staining indicated that a subset of mitovesicles contain functional mitochondria and can restore recipient cells without direct cell–cell interactions.

The research on mitovesicles in sepsis is still sparse. However, it is known that extracellular vesicles, including those of mitochondrial origin, increase considerably in sepsis. These extracellular vesicles carry not only functional mitochondria but also mitochondrial components like mitochondrial DNA, which contribute to the regulation of inflammatory response [70].

### Tunneling nanotube

**Fig. 6:**
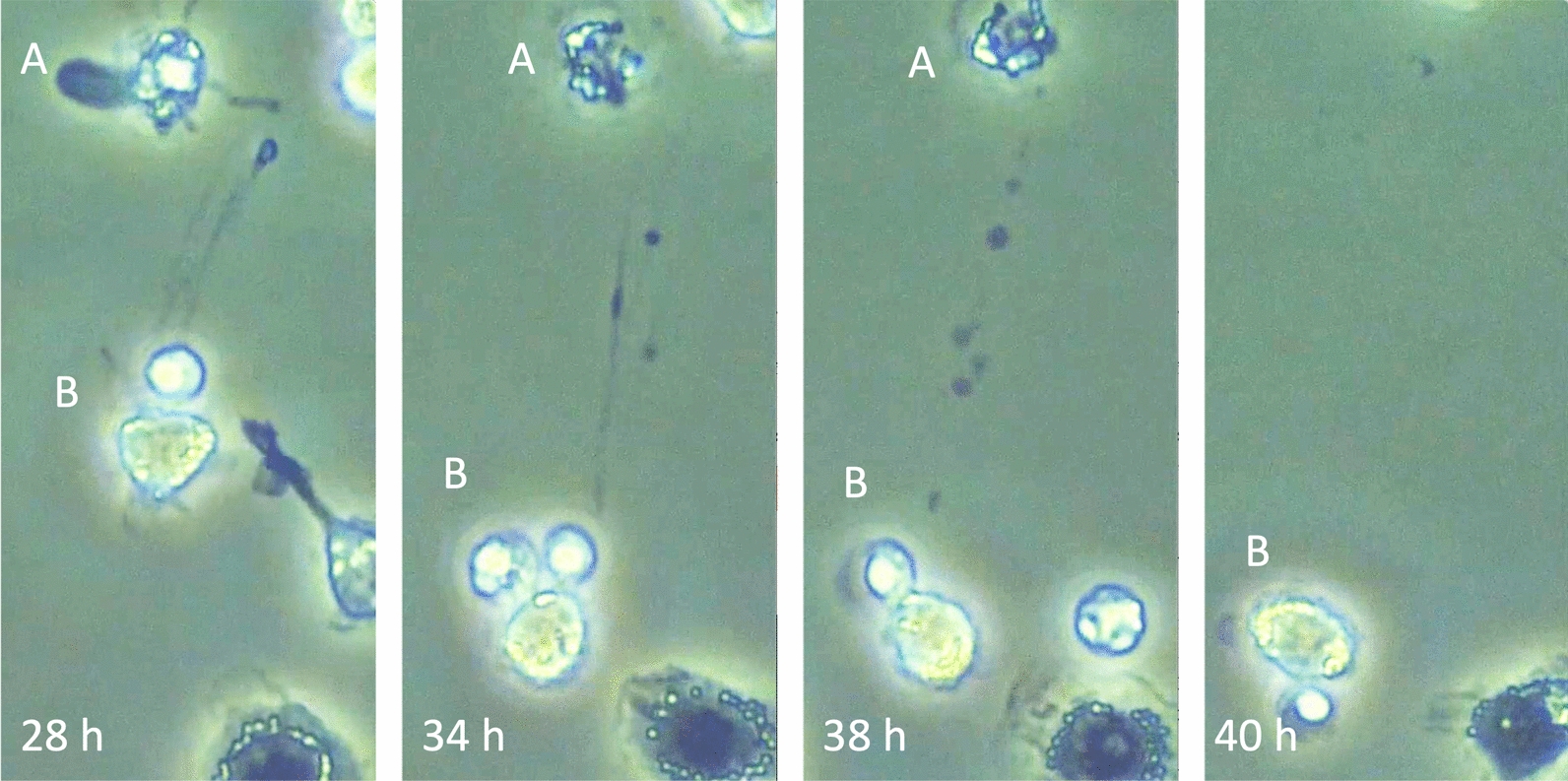
Organelles transport through tunneling nanotube. The image shows leukocytes cultured in a medium without fetal bovine serum. Cell A was significantly damaged, and cell B began to extend a tunneling nanotube at 28 h. The tunneling nanotube attached to cell A at 34 h. Organelles were transported through the tunneling nanotube from cell B to cell A at 38 h. After completing the transportation, cell A moved out of the visual field at 40 h

The transfer of mitochondria by tunneling nanotubes (TNTs) is a unique cellular process that involves the direct transport of organelles between cells. TNTs are long, thin, actin-based structures that connect the cytoplasm of two cells, allowing for the exchange of various cellular components, including mitochondria [71] (Fig. 6). TNTs are typically composed of actin filaments and can vary in length from a few micrometers to several hundred micrometers. They have a diameter of about 50–200 nm, wide enough to allow the passage of various cellular components. The transfer can rescue damaged or stressed cells by replenishing their mitochondrial population, thus restoring energy production and metabolic function. Cells under oxidative stress, nutrient deprivation, or other damaging conditions can receive healthy mitochondria from neighboring cells, enhancing their survival and function [72]. In infectious diseases, tunneling nanotubes are reported to help rescue damaged or stressed cells, especially immune cells. This helps maintain the homeostasis of immune function under severe stress, including sepsis [73]. Interestingly, it is also suggested tunneling nanotubes may transfer pro-inflammatory or anti-inflammatory signals between immune cells, affecting the systemic inflammatory response in sepsis. This can potentially exacerbate or mitigate the immune response based on the type of signals exchanged [74].

## Therapeutic implications of regulating autophagy

Understanding the interplay between autophagy, cell death, and inflammation in sepsis can inform therapeutic strategies. For example, enhancing autophagy can help control excessive inflammation and improve immune function. Conversely, inhibiting excessive autophagy may prevent autophagic cell death and tissue damage (Table 2).

**Table 2:** Therapeutic implications of regulating autophagy

Activation of autophagy	Rapamycin	Rapamycin, an mTOR inhibitor, demonstrated a survival benefit in an animal model of sepsis; however, it failed to demonstrate effects on multiple system atrophy [77, 78]
	Taurolidine	Taurolidine, an antibiotic, was shown to protect mice against microbial sepsis. However, its clinical effects have not proven yet [79, 80]
	Nutrients	The effects of omega-3 fatty acids, eicosapentaenoic acid, docosahexaenoic acid, selenium, zinc, vitamin C, and vitamin E have not been proven [81, 82]
	Permissive underfeeding	Underfeeding can upregulate autophagy. Although its benefit on sepsis is not clear, ASPEN suggests high-protein hypocaloric feeding for obese ICU patients [82]
Suppression of autophagy	Chloroquine Hydroxychloroquine	These agents inhibit autophagosome fusion with lysosomes. The effects of chloroquine on bacterial sepsis are unclear, and its effect on COVID-19 was denied [87, 89]

ASPEN: American Society for Parenteral and Enteral Nutrition

### Enhancement of xenophagy

Autophagy helps eliminate intracellular pathogens through xenophagy. Therefore, modulating autophagy can enhance the clearance of pathogens such as *Mycobacterium tuberculosis* and *Salmonella typhimurium* [75]. Chemicals that stimulate autophagy might be adjunct therapies in infectious diseases. Lee et al. developed chemical mimics of the N-terminal degrons (N-degrons) Nt-arginine to induce targeted degradation of intracellular bacteria via autophagy [76].

Although clinical trials of mTOR inhibitors have not been done for patients with sepsis, the effect of rapamycin was examined in patients with multiple system atrophy. Consequently, the treatment of rapamycin for 48 weeks was futile but had no effect compared to placebo, and this randomized controlled study was terminated after a pre-planned interim analysis met futility criteria [77]. In the experimental model of sepsis, rapamycin reduced inflammation, limited organ damage, and improved survival [78].

Taurolidine is an antibiotic used to prevent catheter-related infections. It is also known to induce autophagy [79]. Huang et al. [80] reported that taurolidine protects mice against microbial sepsis. However, reports on human studies are still lacking.

### Anti-inflammatory nutrition and agents

Targeting autophagy regulation with anti-inflammatory nutrients may help balance the immune response and decrease the severity of sepsis. It has been reported that omega-3 fatty acids attenuate organ damage via AMPK-mediated autophagy activation [81]. However, since clinical studies could not prove the benefit, and the American Society for Parenteral and Enteral Nutrition (ASPEN) does not recommend the routine use of an enteral formulation characterized by an anti-inflammatory lipid profile (e.g., omega-3 fatty acid, eicosapentaenoic acid, and docosahexaenoic acid) and antioxidants (e.g., selenium, zinc, vitamin C, and vitamin E) for critically ill patients [82].

Targeting inflammatory pathways with anti-inflammatory drugs or cytokine inhibitors will be another choice to help break the cycle of autophagic cell death and inflammation, improving outcomes in septic patients. Heme oxygenase-1 (HO-1) contributed to general anti-inflammation. Shutong et al. [83] revealed that increased HO-1 expression inhibited the level of NLRP3 inflammasome via regulating the activation of autophagic flux, thus attenuating inflammatory response and alleviating sepsis-induced acute lung injury.

### Suppression of autophagy

Modulating autophagy is expected to control excessive inflammation and improve immune function during infections. Agents like chloroquine and hydroxychloroquine inhibit autophagy by impairing autophagosome fusion with lysosomes [84]. It is odd to think that inhibiting autophagy can aid in killing bacteria. However, since some studies have reported that macrophages deficient in autophagy exhibit increased uptake of bacteria, inhibiting autophagy with chloroquine may protect against bacterial infection [85, 86]. Lu et al. [87] reported that hydroxychloroquine protected mice against *E. coli* infection-induced lethality by reducing cytokine production and vascular leakage and enhancing bacterial clearance. The immunomodulatory effects of chloroquine and hydroxychloroquine have recently attracted attention to treat COVID-19 [88]. However, the meta-analysis results concluded hydroxychloroquine for COVID‐19 had little or no effect [89].

In conclusion, depending on the context and target organs, both autophagy activation and inhibition can play therapeutic roles in sepsis. Enhancing autophagy often provides protective effects, while inhibition can help control excessive inflammation.

### Permissive underfeeding

Since autophagy is a physiological response to fasting, it is possible to upregulate autophagy by underfeeding. Arabi et al. [90] demonstrated that enteral feeding with a saving of nonprotein calories was not associated with lower mortality in adult critically ill patients. Following this study, the same group reported that permissive underfeeding with full protein intake was associated with similar outcomes as standard feeding among patients with high and low nutritional risk [91].

It has been shown that providing resting energy expenditure early in critical illness does not improve clinical outcomes [92]. Producing sufficient energy from nutrients requires the use of the mitochondrial electron transport chain, which is linked to the creation of ROS. In healthy conditions, the functional activity of ROS is controlled by its production and antioxidants; however, increased production of ROS overcomes antioxidant systems and promotes inflammation in sepsis [93]. Feeding attenuates autophagy and facilitates this process, which has been associated with worse clinical outcomes. ASPEN suggests that high-protein hypocaloric feeding be implemented in the care of obese ICU patients [82].

## Conclusion

In sepsis, the relationships between autophagy, cell death, and inflammation are critical determinants of disease outcomes. Autophagy plays a protective role by modulating inflammation and promoting cell survival, but dysregulated autophagy can lead to exacerbated inflammation and excessive cell death. The type of cell death that predominates in sepsis—whether apoptosis, necrosis, other proinflammatory programmed cell deaths, or autophagic cell death—is related mainly to autophagy and significantly impacts the inflammatory response and overall disease progression. Therapeutic strategies that modulate the above processes promise to improve outcomes in septic patients.

## Data Availability

Not applicable.
